# The sperm-specific K^+^ channel Slo3 is inhibited by albumin and steroids contained in reproductive fluids

**DOI:** 10.3389/fcell.2024.1275116

**Published:** 2024-08-29

**Authors:** Johannes Lorenz, Clara Eisenhardt, Teresa Mittermair, Alexandra E. Kulle, Paul Martin Holterhus, Manfred Fobker, Wolfgang Boenigk, Verena Nordhoff, Hermann M. Behre, Timo Strünker, Christoph Brenker

**Affiliations:** ^1^ Centre of Reproductive Medicine and Andrology, University Hospital Münster, University of Münster, Münster, Germany; ^2^ Division of Pediatric Endocrinology and Diabetes, Department of Pediatrics, Christian-Albrechts-University, Kiel, Germany; ^3^ Center for Laboratory Medicine, University Hospital, Münster, Germany; ^4^ Max Planck Institute for Neurobiology of Behaviour—Caesar, Bonn, Germany; ^5^ Fertility Centre, University Hospital Münster, Münster, Germany

**Keywords:** human sperm, ion channel, sperm signalling, reproductive tract, follicular fluid

## Abstract

To locate and fertilize the egg, sperm probe the varying microenvironment prevailing at different stages during their journey across the female genital tract. To this end, they are equipped with a unique repertoire of mostly sperm-specific proteins. In particular, the flagellar Ca^2+^ channel CatSper has come into focus as a polymodal sensor used by human sperm to register ligands released into the female genital tract. Here, we provide the first comprehensive study on the pharmacology of the sperm-specific human Slo3 channel, shedding light on its modulation by reproductive fluids and their constituents. We show that seminal fluid and contained prostaglandins and Zn^2+^ do not affect the channel, whereas human Slo3 is inhibited in a non-genomic fashion by diverse steroids as well as by albumin, which are released into the oviduct along with the egg. This indicates that not only CatSper but also Slo3 harbours promiscuous ligand-binding sites that can accommodate structurally diverse molecules, suggesting that Slo3 is involved in chemosensory signalling in human sperm.

## Introduction

Human sperm encounter an ever changing chemical composition of the environment during their journey through the female reproductive tract. In particular, they are mixed with seminal fluid upon ejaculation, encounter secretions from cells lining the genital tract and surrounding the egg, and also get into contact with follicular fluid that enters the oviduct upon ovulation.

In human sperm, the sperm-specific Ca^2+^ channel CatSper serves as a promiscuous polymodal chemosensor that translates changes in the chemical microenvironment into changes of the intracellular Ca^2+^ concentration and swimming behaviour (Publicover et al., 2008; Brown et al., 2019; Rahban and Nef, 2020; Wang et al., 2021). Thereby, CatSper functions as a central signalling knot that is required for human fertilization (Brown et al., 2018; Brown et al., 2019; Luo et al., 2019; Wang et al., 2021; Young et al., 2024). In fact, human CatSper is activated in a synergistic fashion (Brenker et al., 2018a) by a plethora of steroids and prostaglandins contained in reproductive fluids (Lishko et al., 2011; Strünker et al., 2011; Brenker et al., 2012; Brown et al., 2017; Mannowetz et al., 2017; Brenker et al., 2018b; Rehfeld, 2020; Jeschke et al., 2021; Wehrli et al., 2023) as well as a bewildering array of synthetic chemicals and compounds used to manipulate enzymes, receptors, and ion channels (Lishko et al., 2011; Strünker et al., 2011; Brenker et al., 2012; Tavares et al., 2013; Schiffer et al., 2014; Rehfeld et al., 2016; Zou et al., 2017; Brenker et al., 2018a; Rennhack et al., 2018; McBrinn et al., 2019; Rehfeld, 2020; Wang et al., 2020a; Zhang et al., 2020; Rahban et al., 2021; Xiang et al., 2022; Torrezan-Nitao et al., 2023; He et al., 2024).

In the sperm flagellum, CatSper is embedded in a network of several ion channels and transporters (Kaupp and Strünker, 2017; Wang et al., 2021), suggesting that chemosensory signal transduction is orchestrated by their mutual interaction (Brown et al., 2019; Wang et al., 2021). A case in point is the sperm-specific Slo3 channel (Schreiber et al., 1998), the principal K^+^ channel in mouse (Navarro et al., 2007; Santi et al., 2010; Zeng et al., 2011; Zeng et al., 2013) and human sperm (Brenker et al., 2014). Such as CatSper, Slo3 is required for sperm function and male fertility (Santi et al., 2010; López-González et al., 2014; Zeng et al., 2015; Lv et al., 2022). Human Slo3 is strongly activated by intracellular Ca^2+^ (Brenker et al., 2014; Geng et al., 2017) and modestly by alkalization (Brenker et al., 2014). Therefore, Slo3 sets the membrane potential of human sperm in a Ca^2+^-dependent fashion (Mannowetz et al., 2013; Brenker et al., 2014), suggesting that Slo3 is involved in chemosensory Ca^2+^ signalling (Kaupp and Strünker, 2017). Supporting this notion, some physiological and synthetic compounds that modulate CatSper were shown to also act on human Slo3. For example, human Slo3 is inhibited by the CatSper agonist progesterone (Brenker et al., 2014; Sánchez-Carranza et al., 2015) and by the CatSper inhibitor RU1968 and derivatives (Rennhack et al., 2018; Schierling et al., 2023), MDL12330A (Brenker et al., 2014), NNC55-0396 (Mansell et al., 2014), and mibefradil (Mansell et al., 2014). This suggests that both human CatSper and human Slo3 harbour ligand-binding sites that can accommodate structurally diverse molecules. However, except for the discovery that the channel is inhibited by progesterone, the pharmacology of human Slo3 regarding physiological ligands encountered by sperm in the male and female reproductive tracts has been understudied. In fact, nothing is known about the action of the various molecules contained in reproductive fluids on human Slo3.

Here, we studied the action of seminal and follicular fluid on heterologously expressed human Slo3. We show that Slo3 is insensitive to seminal fluid and its components prostaglandins and Zn^2+^. By contrast, follicular fluid potently inhibits the channel. We show that human Slo3 is inhibited not only by progesterone, but also by various other steroids of follicular fluid, demonstrating that the channel harbours a promiscuous steroid-binding site. Slo3 is also inhibited by albumin, which is contained in follicular fluid in high micromolar concentrations. In fact, the inhibitory action of follicular fluid on the channel rests on a combined action of albumin and steroids. Remarkably, compared to heterologous Slo3, albumin and, thereby, also follicular fluid inhibit native Slo3 in human sperm with much lower potency and/or efficacy. This suggests a rather indirect albumin action on Slo3 that depends on the particular cellular microenvironment, e.g., the lipid composition of the membrane. In summary, we provide the first experimental evidence that the chemosensory signalling pathways employed by human sperm to track down and fertilize the egg might involve complex combined ligand actions on both CatSper and Slo3.

## Results

**FIGURE 1:**
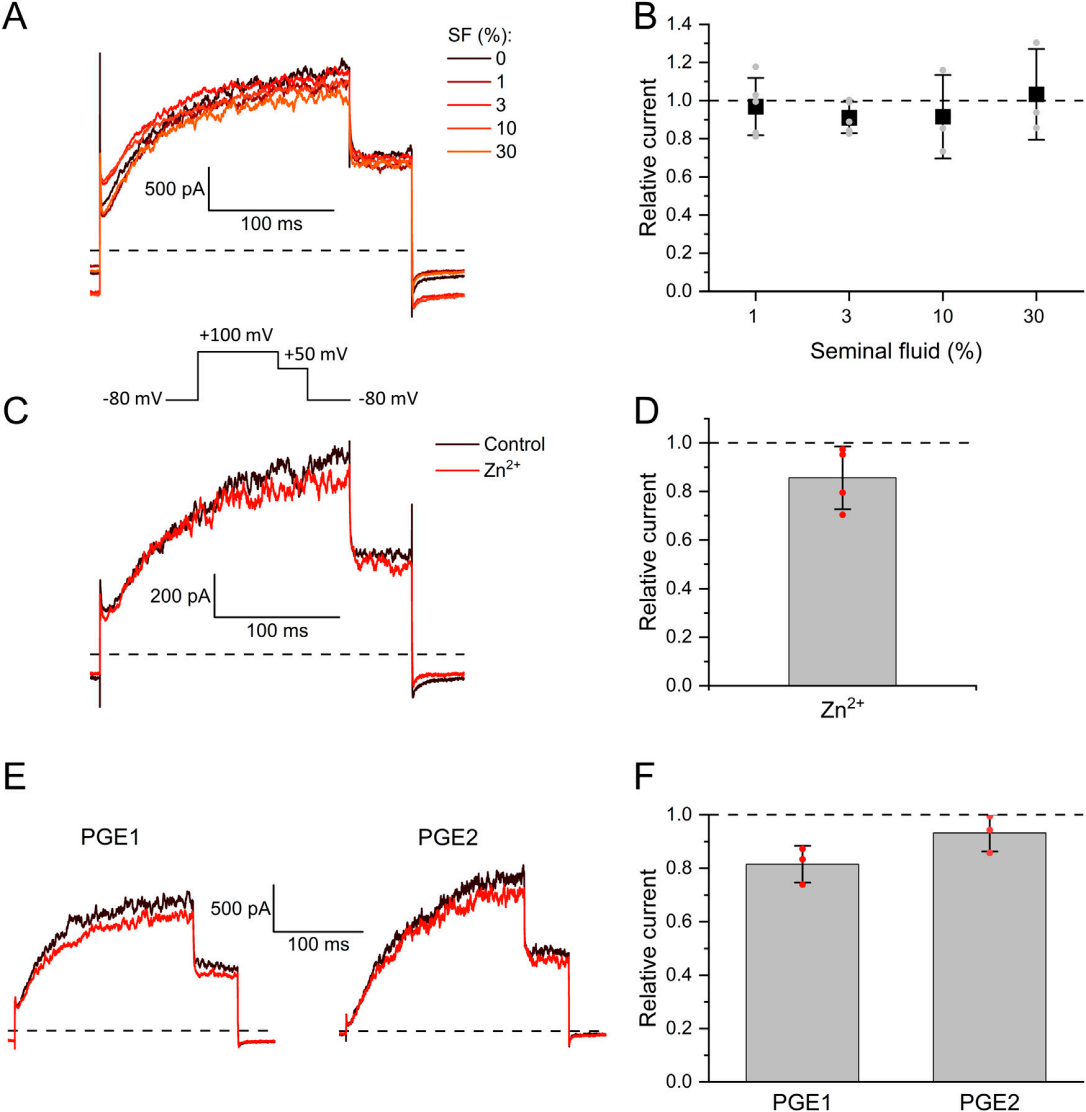
The action of seminal fluid, prostaglandins, and Zn^2+^ on human Slo3 **(A)** Membrane K^+^ currents recorded from CHO cells co-expressing human Slo3 and LRRC52 in the whole-cell configuration before and after perfusion with dilute (%) solutions of seminal fluid (SF). **(B)** Relative current amplitude (mean ± SD) at +100 mV in the presence of a given dilution of seminal fluid relative to that under control conditions (set to 1) (n ≥ 3). Grey dots indicate individual values. **(C)** Slo3 currents before (control, black) and after perfusion with 1 mM Zn^2+^ (red). **(D)** Current amplitudes (mean ± SD) at +100 mV in the presence of 1 mM Zn^2+^ relative to that under control conditions (set to 1) (n = 4). Red dots indicate individual recordings. **(E)** Slo3 currents before (black) and after perfusion with prostaglandin E1 or E2 at a concentration of 50 μM (red). **(F)** Relative current amplitude (mean ± SD) at +100 mV in the presence of 50 µM PGE1/2 (n = 3). Red dots indicate individual recordings.

We investigated the action of seminal and follicular fluid on human Slo3 transiently co-expressed with its auxiliary subunit LRRC52 in CHO cells. We recorded Slo3-mediated currents before and after perfusion with dilute solutions of either fluid and determined the relative change in current amplitude. Slo3 currents did not change upon perfusion with ≤30% seminal fluid (Figures 1A,B), the highest dose tested, indicating that Slo3 is insensitive to its components. Supporting this notion, in contrast to various other ion channels including CatSper (Harrison and Gibbons, 1994; Qiu et al., 2016; Jeschke et al., 2021), Slo3 is not affected by Zn^2+^ (Figures 1C,D), which is contained in seminal fluid at millimolar concentrations. Upon perfusion with 1 mM Zn^2+^, the amplitude of Slo3 currents decreased by 14 ± 13% (n = 4) (Figures 1C,D), which reflects however a general rundown of Slo3 currents over time rather than an inhibitory action of Zn^2+^. We observed a similar slight decrease in Slo3 current amplitudes upon continuous perfusion with control buffer: over 120 s, the current amplitude decreased by 15 ± 6% (n = 6) (Supplementary Figure S1). We also tested the action of individual prostaglandins contained in seminal fluid. Upon perfusion with 50 µM prostagladin E1 (PGE1) or prostaglandin E2 (PGE2), which potently activate human CatSper (Lishko et al., 2011; Strünker et al., 2011; Brenker et al., 2012; Jeschke et al., 2021), current amplitudes decreased only by 18 ± 7% and 7 ± 7% (n = 3) (Figures 1E,F), respectively, which is again in the range of the current rundown. Thus, Slo3 is not affected by prostaglandins either.

**FIGURE 2:**
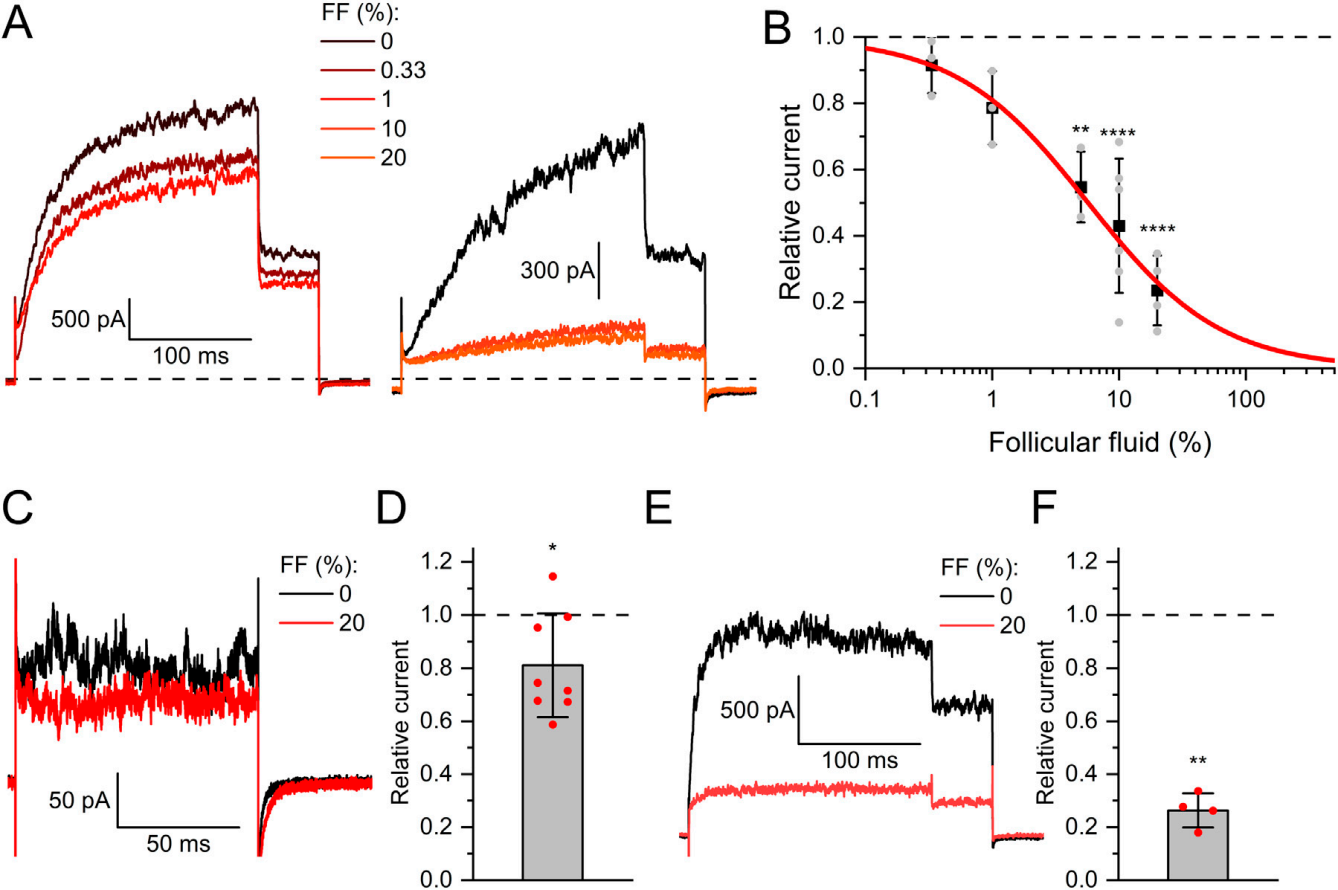
Human Slo3 is inhibited by follicular fluid **(A)** Slo3 currents before and after perfusion with dilute (%) solutions of follicular fluid (FF). **(B)** Current amplitudes (mean ± SD) at +100 mV in the presence of a given dilution of follicular fluid relative to that under control conditions (set to 1) (n ≥ 3). Grey dots indicate individual values. The continuous red line represents a fit of the Hill equation, yielding the dose-response relationship (ID_50_ = 5.7 ± 0.5%, Standard error of the fit). **(C)** Slo3 currents recorded from human sperm at +100 mV before and after perfusion with FF. **(D)** Current amplitudes (mean ± SD) in the presence of FF relative to that under control conditions (set to 1) (n = 8). **(E)** Slo3 currents recorded from CHO cells in the presence of 1 mM intracellular Ca^2+^ and pH 8.0 before and after perfusion with FF. **(F)** Current amplitudes (mean ± SD) in the presence of FF relative to that under control conditions (set to 1) (n = 4). *p < 0.05, **p < 0.01, ****p < 0.0001.

In contrast to seminal fluid, follicular fluid decreased Slo3 currents in a dose-dependent fashion with a half-maximal inhibitory dilution (ID_50_) of 5.7 ± 0.5% (Figures 2A,B). Of note, the fluid is retrieved as a byproduct during ovum pick-up for medically assisted reproduction, which involves its dilution with a flushing medium. The flushing medium itself did, however, not affect Slo3 (Supplementary Figure S2A). Remarkably, follicular fluid inhibited native Slo3 in human sperm with much lower potency and/or efficacy. Perfusion with 20% follicular fluid decreased Slo3 currents in human sperm only by 21 ± 19% (Figures 2C,D), which is in line with previous reports (Brown et al., 2017). Yet, in human sperm, but not in CHO cells, we performed the recordings at high intracellular Ca^2+^ to enhance Slo3 currents (Mannowetz et al., 2013; Brenker et al., 2014) and suppress confounding K^+^ outward currents carried by CatSper at very positive voltages (Zeng et al., 2013; Brenker et al., 2014). We wondered whether Ca^2+^- and/or pH-activation of Slo3 affects the action of follicular fluid. However, in CHO cells, follicular fluid inhibited Slo3 currents in a similar fashion in the absence and presence of intracellular Ca^2+^ and elevated pH (compare Figures 2B and F).

**FIGURE 3:**
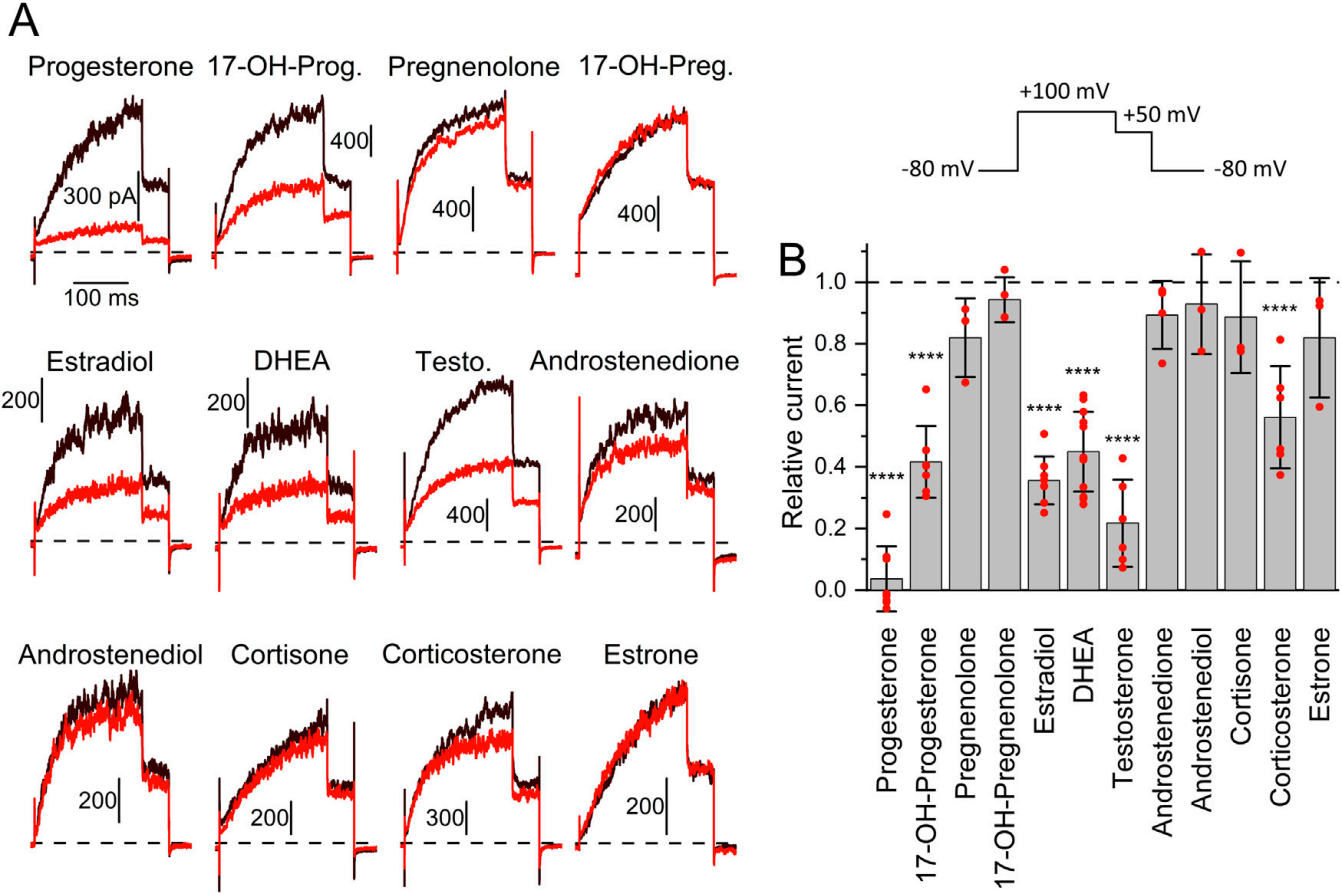
The action of steroids contained in reproductive fluids on human Slo3 **(A)** Slo3 currents before (black) and after perfusion with a given steroid (50 μM, red). (17-OH-Prog. = 17α-Hydroxyprogesterone, 17-OH-Preg. = 17α-Hydroxypregnenolone, DHEA = Dehydroepiandrosterone, Testo. = Testosterone). Vertical and horizontal scale bars represent pA and ms, respectively. Top right: Voltage protocol used for all recordings. **(B)** Current amplitude (mean ± SD) at +100 mV in the presence of 50 µM of the respective steroid relative to that under control conditions (set to 1) (n ≥ 3). Red dots indicate individual values; ****p < 0.0001.

**FIGURE 4:**
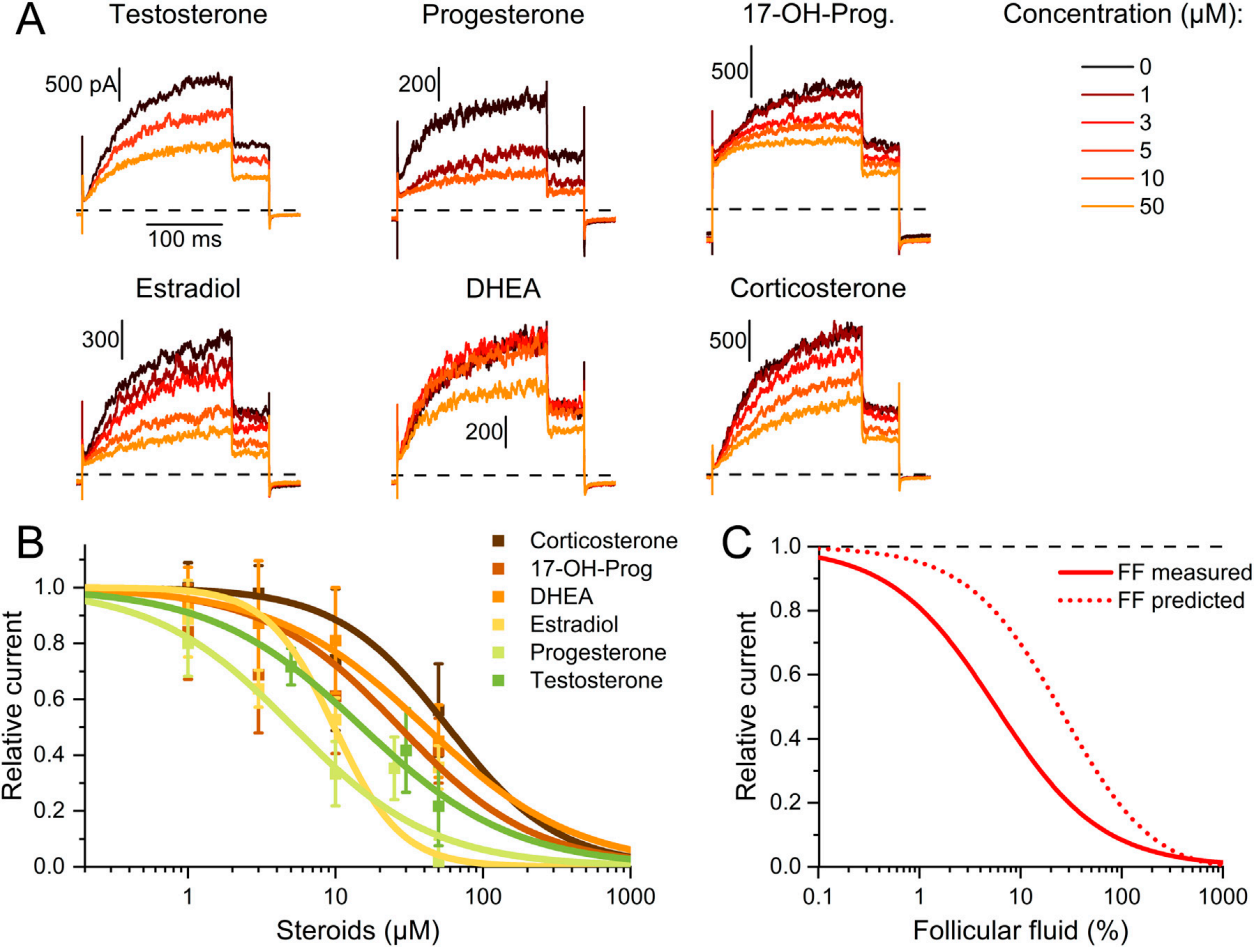
Dose-response relationship for the steroid inhibition of human Slo3 **(A)** Slo3 currents before and after perfusion with different concentrations of a given steroid. **(B)** Current amplitudes (mean ± SD) at +100 mV in the presence of different concentrations of the respective steroid relative to that under control conditions (set to 1) (n ≥ 3). Continuous lines represent fits of the Hill equation to yield the dose-response relationships (n ≥ 3). **(C)** Experimentally determined dose-response relationship of the Slo3 inhibition by follicular fluid shown in Figure 2B (FF, continuous line) and modelled dose-response relationship (dotted line) assuming that its action exclusively rests on the contained steroids (see explanation in the text).

To elucidate the mechanism underlying the different action of follicular fluid on heterologous and native Slo3 in CHO cells and sperm, respectively, we set out to reveal the identity of the molecules acting on the channel. We and others have shown that human Slo3 is inhibited by progesterone (Brenker et al., 2014; Sánchez-Carranza et al., 2015), which is contained in high micromolar concentrations in follicular fluid along with various other steroids (Munuce et al., 2006; Kushnir et al., 2009; Marchiani et al., 2020; Jeschke et al., 2021). This suggests that the inhibition of heterologous Slo3 by follicular fluid is mediated by steroids. To test this hypothesis, we studied the action of the twelve most abundant steroids in follicular fluid on Slo3 (Jeschke et al., 2021). At the initial test concentration of 50 μM, six of the steroids did not or only slightly inhibit Slo3 (Figures 3A,B); perfusion with pregnenolone, 17α-hydroxypregnenolone, androstenedione, androstenediol, cortisone, or estrone decreased the current amplitude only by ≤18% (Figure 2). We thus did not further investigate the action of these steroids. In contrast, progesterone, 17α-hydroxyprogesterone, estradiol, dehydroepiandrosterone (DHEA), testosterone, and corticosterone significantly decreased Slo3 currents (Figures 3A,B); comparison of the current-voltage (IV) relations in the absence and presence of the steroids indicates that the steroid-inhibition features no voltage-dependence (Supplementary Figure S3). We studied the action of these steroids in a dose-dependent fashion (Figures 4A,B). This revealed that the potency of the steroids to inhibit Slo3 follows the sequence progesterone (IC_50_ = 5.2 ± 2.9 μM) > estradiol (8.9 ± 4.4 μM) > testosterone (17 ± 14 μM) > 17α-hydroxyprogesterone (27 ± 10 μM) > DHEA (41 ± 4 μM) > corticosterone (58 ± 11 μM) (n ≥ 4). Follicular fluid also contains micromolar concentrations of arachidonic acid (Li et al., 2020), a known modulator of several ion channels (Martín et al., 2014) that might also act on Slo3. However, perfusion with 3 µM arachidonic acid did not affect Slo3 currents (Supplementary Figure S4), indicating that the channel is insensitive to this molecule.

**FIGURE 5:**
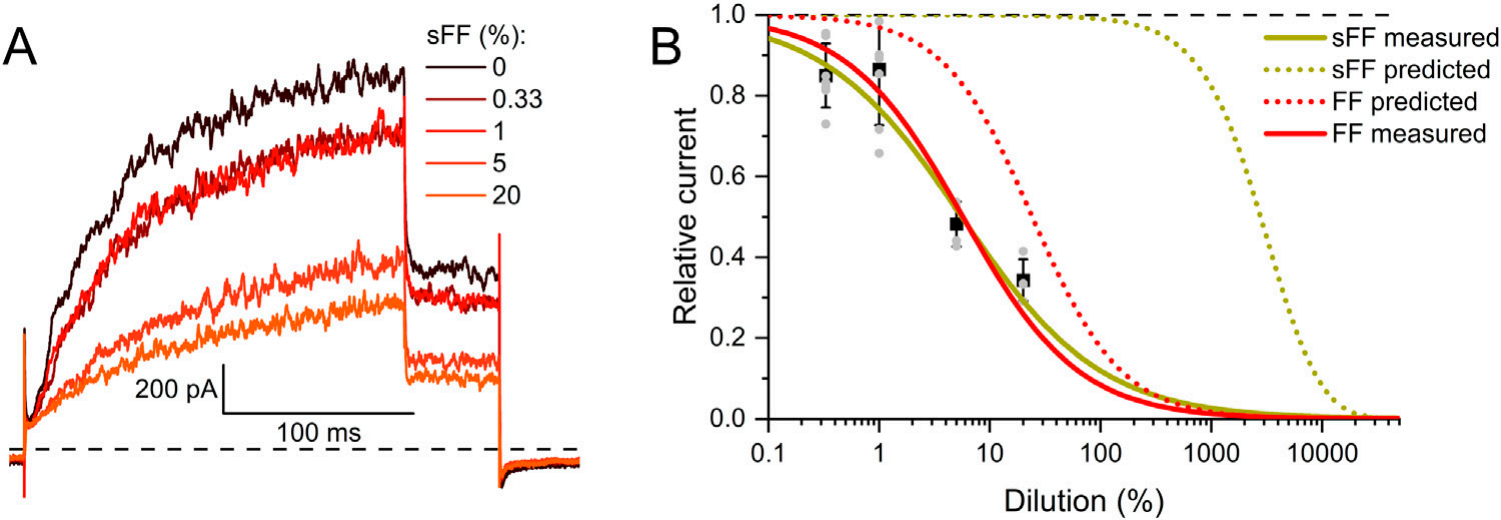
Human Slo3 is inhibited by charcoal-stripped follicular fluid **(A)** Slo3 currents before and after perfusion with dilute (%) solutions of charcoal-stripped follicular fluid (sFF). **(B)** Current amplitudes (mean ± SD) at +100 mV in the presence of a given dilution of sFF relative to that under control conditions (set to 1) (n ≥ 3). Grey dots indicate individual values. The continuous green line represents a fit of the Hill equation, yielding the dose-response relationship (ID_50_ = 5.7 ± 1.3%, Standard error of the fit). For comparison, the dose-response relation for FF from Figure 2B is shown in red. Additionally, the predicted dose-response relations based on the steroid concentrations determined in FF (dotted red) and sFF (dotted green) are shown.

Thus, we wondered whether the inhibition of heterologous Slo3 by follicular fluid might exclusively rest on the action of the steroids. Considering the concentration of the steroids in follicular fluid (Jeschke et al., 2021) as well as their IC_50_ values for Slo3 inhibition, and assuming that they act additively, we modelled a dose-response relation predicting their combined action in follicular fluid (Figure 4C). According to this prediction, follicular fluid should inhibit Slo3 with an ID_50_ of 24.2 ± 0.1%, which is about four times higher than the experimentally determined ID_50_ for Slo3 currents in CHO cells (Figures 2B, 4C). This indicates that the fluid contains molecules other than steroids that also inhibit Slo3. To test for this, we stripped the follicuar fluid of lipophilic molecules using dextran-coated activated charcoal (Beard and Hunter, 1994; Brown et al., 2017), which reduced the concentrations of the individual steroids by up to two orders of magnitude (Table 1). Considering the steroid concentrations in charcoal-stripped follicular fluid and assuming that its action would exclusively rest on these steroids, the charcoal-stripped fluid should inhibit Slo3 with an ID_50_ of 2744 ± 12%. (Figure 5B), i.e., it would have to be concentrated to exert a sizeable action. The experimentally determined ID_50_ values of native and stripped fluid to inhibit Slo3 were, however, similar (5.7 ± 0.5% *versus* 5.7 ± 1.3%) (Figures 5A,B). This indicates that the inhibitory action of follicular fluid on heterologous Slo3 is predominantly mediated by as yet unknown molecules rather than steroids.

**TABLE 1:** Concentration (in nM) of selected steroids in charcoal-stripped follicular fluid (sFF).

	sFF
Progesterone	262
Testosterone	0.32
Estradiol	54
17-OH-Prog.	94
Androstenedione	0.365

**FIGURE 6:**
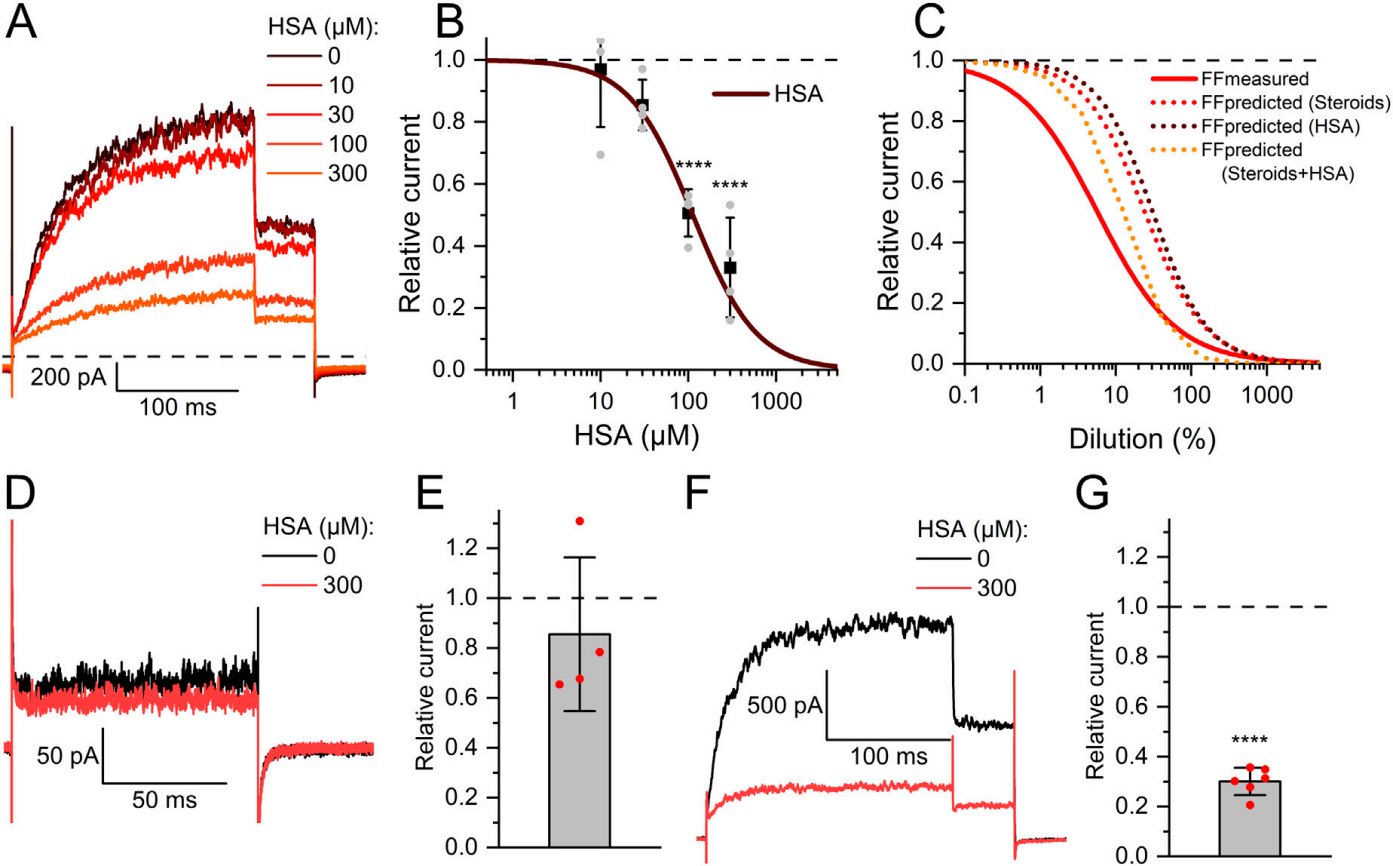
Human serum albumin inhibits human Slo3 **(A)** Slo3 currents before and after perfusion with different concentrations of albumin. **(B)** Current amplitudes (mean ± SD) at +100 mV in the presence of given concentration of albumin relative to that under control conditions (set to 1) (n = 4). The continuous line represents a fit of the Hill equation to yield the dose-response relationship. Grey dots indicate individual recordings. **(C)** Comparison of measured (continous red line) and predicted dose-response relations for FF based on the contained concentrations of steroids (dotted red line), HSA (dotted brown line), or both (dotted orange line). **(D)** Slo3 currents recorded from human sperm at +100 mV before and after perfusion with albumin. **(E)** Current amplitudes (mean ± SD) in the presence of albumin relative to that under control conditions (set to 1) (n = 4). **(F)** Slo3 currents recorded from CHO cells in the presence of 1 mM intracellular Ca^2+^ and pH 8.0 before and after perfusion with albumin. **(G)** Current amplitudes (mean ± SD) in the presence of albumin relative to that under control conditions (set to 1) (n = 6); ****p < 0.0001.

**FIGURE 7:**
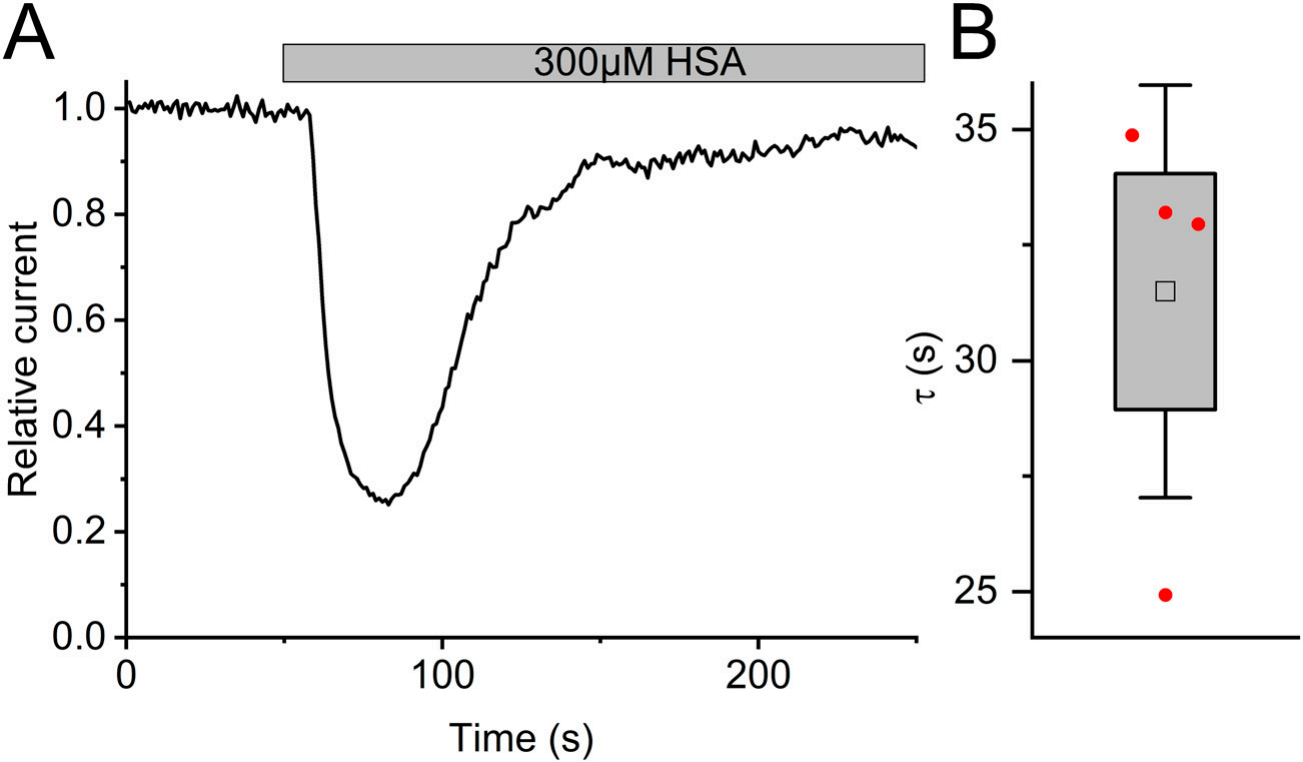
The action of albumin on Slo3 channels is transient **(A)** Time course of the inhibition of Slo3 channels recorded from CHO cells after perfusion with albumin. **(B)** Time constant of the relieve of inhibition in the presence of albumin.

In fact, reproductive fluids also contain albumin in high micromolar concentrations (Casslén and Nilsson, 1984). Therefore, we investigated the action of human serum albumin on Slo3 (Figure 6A). Albumin indeed inhibited Slo3 in a dose-dependent fashion with an IC_50_ of 131 ± 20 μM (n = 4) (Figure 6B). We wondered whether the inhibition of Slo3 by follicular fluid might rest on the contained albumin. To this end, we determined its albumin content. By colorimetric and immunological detection, we determined albumin concentrations of 363 and 343 μM, respectively, matching with previously reported values (Casslén and Nilsson, 1984; Munuce et al., 2006). Next, we modelled the dose-response relation for the action of follicular fluid on Slo3, assuming that its action exclusively rests on albumin (Figure 6C). If true, follicular fluid should inhibit Slo3 with an ID_50_ value of 36% (Figure 6C), which is about fourfold higher than the experimentally determined value and, thus, in the same range as the ID_50_ value predicted for an action based exclusively on steroids. Importantly, in contrast to steroids, charcoal stripping of follicular fluid did not affect the albumin content, i.e., its concentration in the stripped fluid was still 330 µM. This finding indicates that the inhibition of heterologous Slo3 by follicular fluid rests on the action of albumin rather than of steroids. However, modelling of the dose-response relation for the action of follicular fluid on Slo3 based on a combined action of steroids and albumin (Figure 6C) predicted an ID_50_ value of 10.8%, i.e., close to the experimentally determined value, suggesting that in fact, inhibition of heterologously expressed Slo3 channels by follicular fluid involves a combined action of steroids and albumin. It is well-established that steroids, i.e., progesterone, affect heterologous and native human Slo3 in a similar fashion (Mannowetz et al., 2013; Brenker et al., 2014; Sánchez-Carranza et al., 2015; Brown et al., 2017). This indicates that the greatly reduced potency/efficacy of follicular fluid to inhibit Slo3 in sperm is not due to a reduced steroid sensitivity of the native *versus* heterologous channel. Therefore, we tested whether Slo3 in human sperm might be less sensitive to albumin. Indeed, 300 µM albumin decreased Slo3 currents in human sperm, if at all, only slightly by 15 ± 30% (n = 4) (Figures 6D,E); the decrease in amplitude was statistically not significant. We wondered whether this might be due to the high intracellular Ca^2+^ concentration used for recordings from sperm. But, albumin also decreased Slo3 currents in CHO cells by 70 ± 5% (n = 6) at both high intracellular Ca^2+^ and elevated pH (Figures 6F,G). Thus, we conclude that in human sperm, Slo3 is much less sensitive to albumin, which explains the different action of follicular fluid on Slo3 in sperm *versus* CHO cells. To gain insight into the mechanism underlying the more potent and/or efficacious inhibition of heterologous Slo3 by albumin, we investigated its action in a time-resolved fashion using repetetive-pulse protocols. This revealed that the inhibition rapidly evolves and peaks within 10–20 s. Surprisingly, the channel showed a pronounced desensitization to albumin-inhibition, i.e., despite the continuous presence of albumin, the currents slowly recovered with a time constant of 31.5 ± 4.5 s (n = 4) until the amplitude settled on a level only slightly below that recorded before perfusion with albumin (Figure 7). The transient inhibition of Slo3 in CHO cells, but not in sperm, suggests an indirect rather than direct action of albumin on the channel, which might be masked under the conditions that we used to record from human sperm. Nevertheless, altogether, our results demonstrate that human Slo3 is modulated by various molecules that are released into the female genital tract, suggesting that the channel is involved in chemosensory signalling pathways employed by human sperm to track down and fertilize the egg.

## Discussion

This comprehensive study on the pharmacology of the sperm-specific human Slo3 channel sheds light on its modulation by reproductive fluids and their constituents. We show that the channel is not affected by molecules in seminal fluid including prostaglandins and Zn^2+^, but potently inhibited by follicular fluid and its constituents. We demonstrated that several steroids in follicular fluid inhibit Slo3, yet, with different potency. Although the number of steroids tested is not sufficient to derive a detailed structure-activity relationship, it is unequivocal that the pharmacology of the steroid action on Slo3 and CatSper is distinctively different. For example, while progesterone and 17-OH-progesterone activate CatSper with similar potency and efficacy (Strünker et al., 2011; Jeschke et al., 2021), the addition of an OH group at the 17α-position of progesterone renders 17-OH-progesterone 5-fold less potent for its action on Slo3. Moreover, while estradiol is around 100-fold less potent than progesterone to activate CatSper, the steroids inhibit Slo3 with similar potency.

The steroid inhibition of Slo3 is another example of non-genomic steroid actions on ion channels, which control a variety of cellular functions. For example, TRPM3 channels are activated by steroids and, thereby, promote insulin secretion in pancreatic ß-cells (Wagner et al., 2008), Kv4.2 act as steroid sensors in granulosa cells (Kunz et al., 2006), and modulation of KIR7.1 channels is thought to regulate epithelial function (Björkgren et al., 2021). In some cases, the molecular mechanism of steroid action has already been elucidated. Slo1 channels, for example, are modulated by cholane steroids only in the presence of ß1-accessory subunits, which harbour three unique amino acids that are essential for steroid binding (Bukiya et al., 2011), whereas cortisone dissociates K^+^ channels from the Shaker family from their accessory β subunits, relieving the channel from N-type inactivation (Pan et al., 2008). Progesterone also inhibits rat (Wang et al., 2020b), but not mouse Slo3 (Mannowetz et al., 2013). The steroid sensitivity of Slo3 in other species, e.g., bovine (Schreiber et al., 1998), remains to be determined. With almost certainty, steroids bind directly to the Slo3-channel complex rather than to a so far unknown steroid-binding protein associated with it, considering that we observe the steroid action on heterologous Slo3/LRRC52 channels in CHO cells. Whether the steroid-binding site is located on the channel itself, on its accessory subunit, or formed at their interface remains to be elucidated. Thus, investigating the steroid action on human Slo3 co-expressed with mouse LRRC52 and vice versa might shed light on the mechanism of action. To investigate this further, steroid photo-affinity labels might serve as powerful tools to identify the residues that bind the steroids. Alternatively, homology modelling of Slo3’s structure based on the Slo1 structure (Tao and MacKinnon, 2019) could be performed to identify putative binding sites on human Slo3; ensuing molecular modelling might allow to virtually probe and functionally scrutinize the pharmacology of putative binding sites.

We show that human Slo3 heterologously expressed in CHO cells is not only modulated by steroids, but also by albumin at physiological concentrations. The protein acts on ion channels either by direct binding to the channel complex (Zhao et al., 2021) or indirectly by depletion of particular lipids from the cell membrane (Sankaranarayanan et al., 2013; Bukiya and Dopico, 2019; Han et al., 2022). We propose that the albumin action on Slo3 rests on the latter. The transience of its action suggests that albumin depletes an as yet unknown messenger molecule, e.g., a membrane lipid, interacting with Slo3, which is slowly replenished over time, causing the relieve from inhibition. In human sperm, under the conditions used here, this particular control mechanism of Slo3 seems lacking. This may be due to the particular lipid content in the flagellar plasma membrane. For example, the level of PtdIns(4,5)P2 (PIP2) is much lower in the membrane of sperm flagella compared to other cells (Kawai et al., 2019). Slo3 is well adapted to this lipid environment: compared to channels in somatic cells, Slo3 is much more sensitive to PIP2 (Tang et al., 2010; Kawai and Okamura, 2022). Future studies are required to test the hypothesis of a lipid-mediated albumin action on Slo3 and, if true, unravel the underlying mechanism, e.g., by analyzing changes in the lipid content of CHO cells under control conditions and upon perfusion with albumin. This might then allow to investigate in a targeted approach the physiological role of this lipid-modulation of Slo3 in sperm. Moreover, such an approach would also enable to test whether this modulation is similar in Slo3 from different species or specific for human Slo3. Alternatively, human sperm might harbor so far unknown Slo3-associated proteins that control the sensitivity of the channel to albumin. Such a cell-specific tuning of channel properties is well-known for Slo1 channels (Gonzalez-Perez and Lingle, 2019).

Our finding that components of follicular fluid inhibit Slo3 adds to the complexity of chemosensory signalling in sperm. We and others have shown before, that follicular fluid activates CatSper in human sperm (Brown et al., 2017; Jeschke et al., 2021). Moreover, it was shown that albumin activates Hv1 channels (Zhao et al., 2021) that are also expressed in human sperm (Lishko et al., 2010; Berger et al., 2017) and, thereby, alkalizes their intracellular pH (Zhao et al., 2021). These findings indicate that follicular fluid engages multiple signalling events in human sperm. Thus, to gain further insights into the molecular makeup and orchestration of the signal transduction pathways in human sperm, kinetic multiplexed recordings of intracellular Ca^2+^, pH, and the membrane potential are required, using, for example, frequency- and spectrally-tuned multiplexing of fluorescent probes (FAST^M^) (Kierzek et al., 2021) to investigate the chemosensory signal flow. The use of specific inhibitors for Slo3 channels (Lyon et al., 2023; Zhang et al., 2024) might allow to disentangle its role in chemosensory signalling.

Finally, an important question concerns the role of the action of steroids and albumin in follicular fluid on CatSper, Slo3, and Hv1 in human sperm during the fertilization process. In fact, the fluid gets diluted quickly after ovulation and only small amounts enter the oviduct (Hansen et al., 1991). It has been proposed that in the oviduct, sperm get into contact with fluid diluted to 0.5% (Hansen et al., 1991), which would still be sufficient to activate CatSper, but not to inhibit and activate Slo3 and Hv1, respectively. However, also uterine fluids contain albumin and steroids in concentrations similar to those determined in follicular fluid (Casslén and Nilsson, 1984; Libersky and Boatman, 1995; Munuce et al., 2006; Lamy et al., 2016). Unfortunately, the exact composition of these fluids is unknown and might vary among species. Thus, quantitative analyses of reproductive fluids especially in humans are required to gain further insights into the ligand control of sperm behaviour during fertilization.

## Materials and methods

### Chemicals

Steroids and prostaglandins were purchased from Sigma-Aldrich and Cayman Chemical and dissolved in DMSO at a concentration of 20 mM. These stock solutions were then diluted in extracellular solution (ES, see below) to the final concentration.

### Reproductive fluids

Follicular Fluid (FF) and seminal fluid (SF) were collected from females undergoing hormonal stimulation for assisted reproduction at the Fertility Centre of the University Hospital in Münster and from “healthy” male donors, respectively, as previously described (Jeschke et al., 2021), with prior written informed consent according to the protocols approved by the Ethical Committees of the Medical Association Westfalen-Lippe and the Medical Faculty of the University of Münster (reference numbers: 1IX Greb 1, follicular fluid; 4INie, 2021-402-f-S, seminal fluid) and the Declaration of Helsinki. In brief, FF was collected by transvaginal aspiration during which it was diluted to 66% with flushing medium (Gynemed, GM501 Flush). To remove cellular components, FF was centrifuged at 3000 g for 10 min and the supernatant was used and stored in aliquots at −20°C. SF was extracted from the ejaculate by two centrifugations at 700 g for 20 min to remove cellular components; the supernatant was collected and stored in aliquots at −20°C. FF was stripped (sFF) from lipid mediators using dextran-coated charcoal as described before (Bedu-Addo et al., 2007; Brown et al., 2017). First, charcoal (0.25% w/v, Sigma-Aldrich, C9157) was coated with dextran T70 (0.0025% w/v, Pharmacia LKB, 17-0280-01) by mixing in a saline solution containing 1.5 mM MgCl_2_, 10 mM HEPES and 0.25 M sucrose (adjusted to pH 7.4 with NaOH) and incubated overnight at 4°C. 8 mL of the dextran-coated charcoal was centrifuged and the pellet was resuspended in 4 mL of FF. After incubating overnight at 4°C, the charcoal-FF mixture was centrifuged at 1000 g for 5 min to pellet the charcoal and obtain the supernatant, i.e., sFF. Finally, the sFF was filtered using a 0.22 μm filter. The steroid hormones were determined by LC-MS/MS as described (Kulle et al., 2010; Kulle et al., 2013; Reinehr et al., 2020; Jeschke et al., 2021). Albumin was measured with a colorimetric method (bromocresol purple dye-binding) on a cobas c701 clinic chemistry analyzer (Roche Diagnostics GmbH, Mannheim, Germany) and by an immune-nephelometric method on a BN II system (Siemens Healthcare Diagnostics GmbH, Eschborn, Germany) according to the standard procedure recommended by the manufacturer. Both methods were validated by regular analyses of reference sera supplied by the national German INSTAND proficiency testing program and the international quality assurance program of the US Centers for Disease Control and Prevention.

### Cell culture

CHO cells were cultivated at 37°C, 5% CO_2_, in F-12 medium (gibco F-12 Nut Mix (1x) + GlutaMAX™ (REF: 31765-027, LOT: 2246389) with 10% FBS, 2 mM L-Glutamine and 1% Penicillin-Streptomycin) at a concentration between 2 × 10^6^ - 6 × 10^6^ cells/mL. CHO cells were co-transfected with a pcDNA3.1(+) vector containing the full length coding sequence of human Slo3 (Accession number: NM_001031836) modified with a carboxy-terminal hemagglutinin tag (HA-tag) and in which the sequence coding for the neomycin resistance gene was replaced by the coding sequence for citrine and a pcDNA3.1(+) vector containing a sequence encoding hLRRC52-mCherry (Accession number: NM_001005214) for 6-8 h using Lipofectamine 2000 (Invitrogen) in Opti-MEM (Gibco). DNA was used in concentrations between 2 and 5 ng/μL. After the transfection, cells were cultivated in F-12 medium. 24 h after transfection the cells were transferred onto poly-l-lysine-coated coverslides and stimulated with 5 mM Na-Butyrate at least 12 h before the experiment.

## Electrophysiology

Electrophysiological recordings were performed from Citrin- and mCherry-positive cells in the whole-cell configuration using an Axopatch 200B patch clamp amplifier (Molecular Devices, Sunnyvale, CA, United States) controlled by the Clampex 10.7 software (Molecular Devices). Signals were low-pass filtered at 10 kHz with a four-pole Bessel filter and digitized with a Digidata 1440A data acquisition system (Molecular Devices). A step protocol with steps from −100 mV to +150 mV followed by a step to 50 mV from a holding potential of −80 mV was used. Cells were perfused with extracellular solution (ES; 140 mM NaCl, 5.4 mM KCl, 1 mM MgCl_2_, 1.8 mM CaCl_2_, 5 mM Hepes, 10 mM Glucose, pH 7.4 with NaOH) and the pipette resistance was 4–6 MΩ with the intracellular solution (130 mM K-Aspartate, 10 mM NaCl, 1 mM EGTA, 5 mM HEPES, 15 mM glucose, pH 7.3 with KOH). In some experiments glucose was replaced by additional HEPES. Pipette solution for recordings with elevated [Ca^2+^]_i_ and pH_i_ was (130 mM K-Aspartate, 10 mM NaCl, 20 mM HEPES, 1 mM CaCl_2_, pH 8.0 with KOH). All substances were diluted in ES and applied to the cells by a gravity-driven perfusion system. Experiments were performed at room temperature.

Recordings from swim-up sperm (Young et al., 2024) in the whole-cell configuration were performed as described before (e.g., Strünker et al., 2011; Brenker et al., 2014) according to the protocols approved by the Ethics Committee of the Medical Association Westfalen-Lippe and the Medical Faculty Münster (2021-402-f-S) and the Declaration of Helsinki. Seals between pipette and sperm were formed at the cytoplasmic droplet or neck region in extracellular solution (HS) containing (in mM): 145 NaCl, 5 KCl, 1 MgSO_4_, 2 CaCl_2_, 5 glucose, and 20 HEPES, adjusted to pH 7.4 with NaOH. The pipette (10–15 MΩ) solution contained (in mM): 130 K-aspartate, 5 KCl, 50 HEPES, and 1 CaCl_2_, adjusted to pH 7.3 with KOH. Data were not corrected for liquid junction potentials.

### Data analysis and statistical evaluation

Analysis was performed using Clampfit 10.7 (Molecular Devices) and OriginPro 2020 (OriginLab Corporation, Northampton, MA, United States). The inhibition was calculated based on the currents from the +50 mV pulse following the +100 mV step (voltage-step protocol Figure 1). We corrected for leak current in each measurement by subtracting the current at the +50 mV pulse immediately following a −100 mV step, i.e., before opening of Slo3 commenced. Fits were performed with a modified Hill equation $y=1-\frac{x^{n}}{k^{n}+x^{n}}$ . All results are given as mean ± standard deviation (number of experiments). To calculate the predicted action of a component in follicular fluid, its IC_50_ value was divided by its concentration in follicular fluid to calculate the expected ID_50_ value for the action of follicular fluid based on the respective component. The predicted action of multiple components was calculated by multiplying the hill equations for each individual component. For the calculation of the time course of the relief from inhibition by albumin the data was fitted with a mono-exponential decay starting from the time point of maximal inhibition.

All data are given as mean ± standard deviation. Statistical analysis was performed whenever meaningful, i.e., on data shown in Figures 1–3, 6, using GraphPad Prism 10.2.3 (Prism, La Jolla, United States). The relative changes in current amplitude upon perfusion with the different stimuli were compared to the relative changes in current amplitude upon 30 s perfusion with buffer to account for current rundown (Supplementary Figure S1). We used one-way ANOVA, assuming sphericity. When ANOVA´s F-test and the test for matching efficacy achieved p < 0.05, means were compared to the control´s mean by Dunnett’s multiple comparisons *post hoc* test. For recordings from human sperm or from CHO cells with high intracellular Ca^2+^ and pH_i_, the current amplitudes before and after application of the respective stimulus were analyzed with Student´s paired t-test.

## Data Availability

The original contributions presented in the study are included in the article/Supplementary Material, further inquiries can be directed to the corresponding author.
