# Supplementary material for: The sperm-specific K+ channel Slo3 is inhibited by albumin and steroids contained in reproductive fluids
Source: Front Cell Dev Biol. 2024 Aug 29;12:1275116. doi: 10.3389/fcell.2024.1275116 (PMC11413451; doi:10.3389/fcell.2024.1275116)
Supplement: Supplementary file 1 [file Image1.pdf]

Supplementary Information:

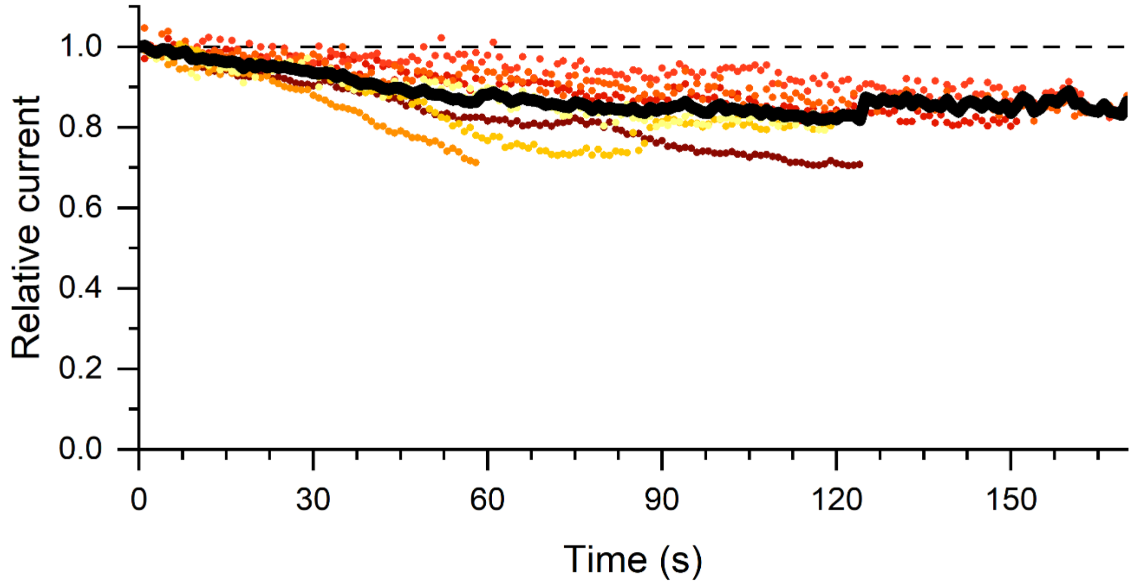

Fig. S1: Rundown of human Slo3 currents. Time course of amplitude of Slo3 currents at +100 mV relative to that after establishment of stable recording conditions, which usually takes around 30 s (set to 1). The black trace represents the average change, the colored dots individual recordings.

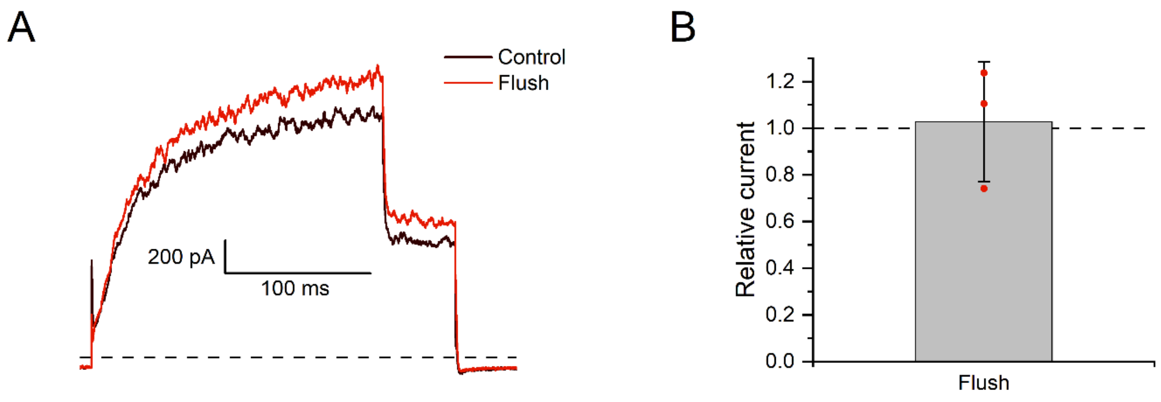

Fig. S2: Flushing medium does not affect human Slo3 (A) Slo3 currents before (control, black) and after perfusion with a 1:1 dilution of flushing medium (flush, red). **(B)** Current amplitudes (mean ± SD) at +100 mV in the presence of the 1:1 dilution of flushing medium relative to that under control conditions (set to 1) (n = 3). Red dots indicate individual recordings.

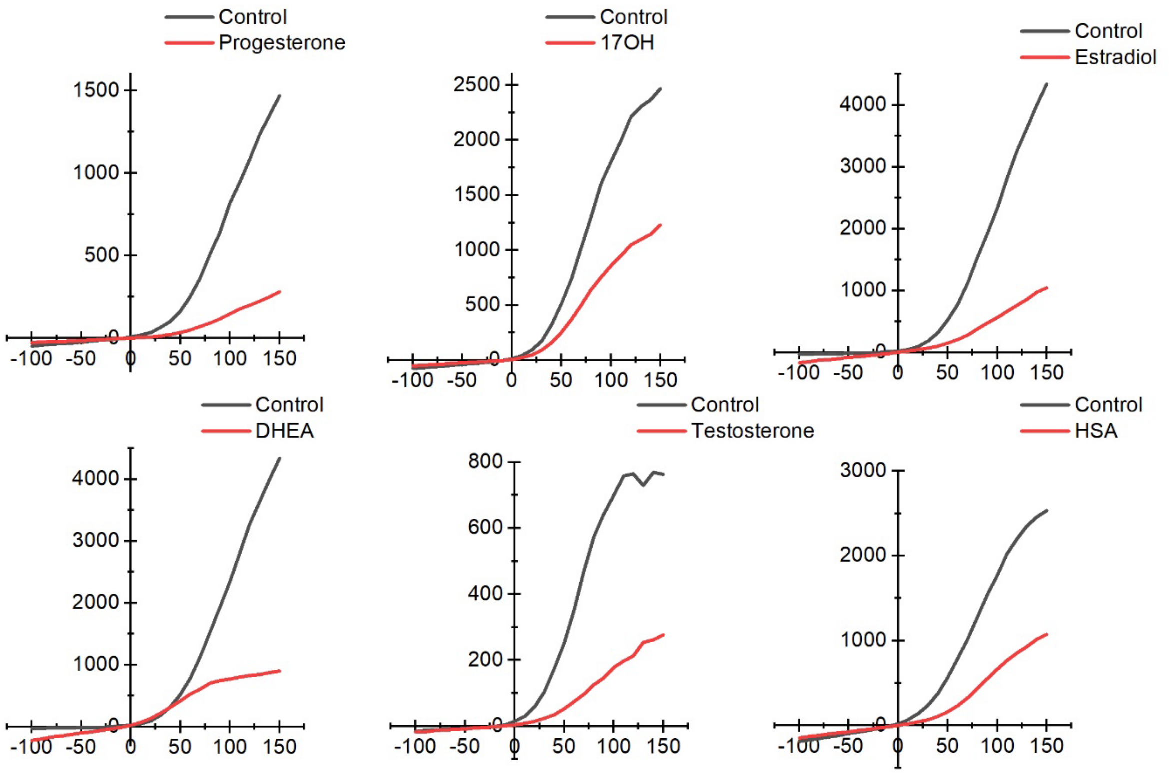

Fig. S3: Inhibition of human Slo3 expressed in CHO cells does not show voltage-dependance. IV relation of Slo3 currents recorded before (control, black) and after perfusion with indicated steroid (50 μM) or albumin (300 μM).

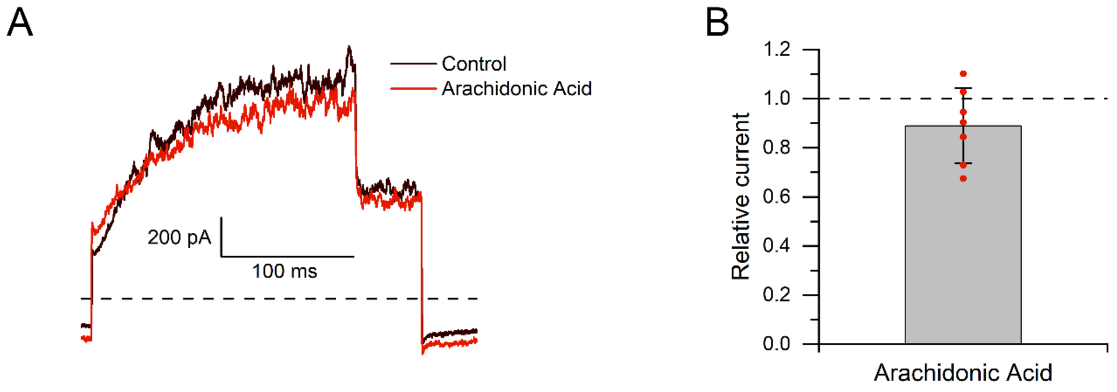

Fig. S4: Arachidonic acid does not affect human Slo3 (A) Slo3 currents before (control, black) and after perfusion with 3 μM arachidonic acid. **(B)** Current amplitudes (mean ± SD) at +100 mV in the presence of 3 μM arachidonic acid relative to that under control conditions (set to 1) (n = 7). Red dots indicate individual recordings.
